# Dental Jurisprudence

**Published:** 1885-03

**Authors:** J. Allen Osmun

**Affiliations:** Newark, New Jersey


### DENTAL JURISPRUDENCE.

READ BEFORE THE SECOND DISTRICT DENTAL SOCIETY OF THE STATE OF NEW YORK.

BY DR. J. ALLEN OSMUN, NEWARK, NEW JERSEY.

MR. PRESIDENT AND GENTLEMEN:

In inviting your attention to this subject I presume that it is one in which you feel a passing interest, although, in the busy days in which you are the chief actors, it may not have enlisted that amount of attention and thought to which the matter is entitled. In fact, I may embrace this occasion to remark that it is a matter of some surprise that it has received so little attention at the hands of our profession. I therefore beg your indulgence while I hastily sketch in rough outlines some of the more prominent reasons why the dental fraternity should have their attention directed thereto. The old Scotch gardener remarked, when asked why he was so particular in protecting his plants when the chances of frost were so light, that he would "rather be sure than sorry."

We must start from the premises, that legal or forensic medicine is the science that teaches the application of medicine to the purposes of law. The grand object of law is the establishment of truth, and for this purpose it levies a tax upon all sources, dental among the rest; and may I be pardoned if I suggest that there are many cases where it draws largely from this source, some of which I may refer to later.

There is, seemingly, a growing spirit of liberalism pervading medical circles, and the lines are converging, which I trust at no far future day will meet, by medical and dental colleges uniting on one common platform, in all that can elevate the one or broaden the other; that it will not be long when we can say, as Madam de Stael said to Napoleon; "Sire, genius knows no sex." So may we affirm that the science of healing knows no "sects."

You are aware, I presume, what progress is being made in this direction by the Medico Legal Society of New York. They are investigating all manner of questions and diseases that pertain to this subject of Medical Jurisprudence, but I have failed to see in any of their published reports any reference to the dental profession, or to questions that affect us in any degree. As their by-laws prohibit any but physicians, lawyers and chemists from membership, this may in part explain the omission.

This society of which I speak, comprises some of the brightest intellects of each of the three professions named, and if they find this field so full of good things, I take it we too can find a few diamonds of knowledge, if we will but search for them.

We must recognize the fact that as professional men, dealing with diseased tissues, we are liable at any time to be put on the defensive from charges of mal-treatment; or that we may be called upon, in the capacity of expert witnesses, to give our professional opinion in courts of law; and as we well know, there is no other way of excluding the testimony of ignorant pretenders or empirics upon professional subjects, or eliciting the truth, except the test of cross-examination, and to be effectual for that purpose it must be allowed to become very searching; and I am convinced that, while it is not always done justly or fairly, it is the only mode of bringing out the facts before the intelligent jury; we do not realize how much need we have for accurate diagnoses, prognoses, and deductions of critical attention to our opinions, until we have them weighed and dissected by counsel before a jury.

There have been times without number when dentists have been in court, either as plaintiff or defendant, in suits of all descriptions. In fact, a superficial observer would be surprised to discover the close relationship existing between the two sciences of law and medicine. Let me recall a few cases where this is true; where life has been lost under the influence of anesthesia; where injuries have been inflicted from unskilled operations; where diseases of the oral cavity have been improperly handled; where testimony has been needed to identify remains from accident or suicide; where serious charges of attempted assaults of a sexual character while under anesthesia have been made; of the question and legality of fees and payment thereof. It were easy to multiply cases in illustration of this nature, but the foregoing will suffice to show something of the importance of this subject.

I am led to suggest that the very first case that a young practitioner could have might involve these very principles, and it, of course, is liable to come to any of us, at any moment.

When called into court as a witness, you will often find that your testimony will be opposed by others engaged by the other party to the suit. Then it will be of great importance to your side if your evidence can be substantiated by indisputable proofs, drawn from sources that admit of no question.

One of the greatest—shall I say the greatest?—attainments of any professioual man, is the cultivation of the faculty of close, minute observation; to be accurate in observation, to be critical in your deductions, is of paramount importance. Nor should it be supposed for a minute that this cultivation of the habit of minute observation will be incompatible with the other duties pertaining to our daily toil; it will, on the other hand, greatly enhance our usefulness.

That we often play an important part in the drama cannot be disputed, and, in the position of professional men, we are often criticised. For instance, Dr. Samuel Sexton, in a series of articles published in the *Medical Record*, said that, "It was believed that since Dentistry had become such a popular business, and diseased teeth had been so carefully retained in the jaws, through their influence, especially among the better-to-do, nervous diseases about the head were becoming alarmingly common. The very custom of wearing false teeth in the mouth attached to vulcanite, rubber, celluloid, and other plates, was also an evil of vast proportions; indeed, he sometimes thought that the evil done through ill-advised dentistry was greater than the possible good arising from the work of the more capable dentists."

This shows you what a position we are placed in. He condemns the leaving of pulpless teeth in the mouth, and then condemns the wearing of substitutes. I admit that he qualifies his statement at the last by saying the "more qualified dentists." The point I desire to bring out is that, if writers of Dr. Sexton's abilities find such charges to make, what are the laity liable to charge, when something has not gone to their entire satisfaction? And if they can bring medical men who would be biased by such articles as the one from which this sentence was quoted, will it not behoove us to be ready to fight fire with fire?

Our testimony should be the colorless light of science, brought to bear upon any case when it is summoned. It should be impartial, unprejudiced; there should be no half truths uttered, but the benefit of the knowledge acquired, perhaps by years of study and observation, should be freely given. Take a case so commonly cited to show how important professional evidence is. Stained garments are placed in the hands of the expert chemist to decide the significance of those stains by scientific tests. His chemical reagents show them to be blood stains. But to leave the matter here might be to utter a falsehood in the name of science. He examines with the microscope, and testifies that the size and shape of the corpuscles prove it to be the blood of a fish or reptile; that it cannot be the blood of a mammalian. He has thus settled the all-important question in the case, and thus his scientific knowledge has solved the vital problem which could not have been determined by a layman, and thereby may have saved a life. So, time and again we shall have opportunity to bring our special knowledge to bear, and by its exercise shield or ward off the impending calamity, or help justice convict the empiric or quack. I will cite a few cases which will tend to show what value is sometimes placed on parts of the human body by juries: A man was struck on the head by an iron poker in the hands of a brakeman ; the external table of his skull was cracked. In consequence of the injury he was threatened with paralysis of the optic nerve. He sued the railway company for the wrong inflicted by their servant, and recovered $4,000.

$10,000 was paid for injury to a boy's brain received by the falling upon him of a berth in a Pullman car.

£500 was given for the injury to the spine of a lady caused by being compelled to jump from a railway carriage.

$7,500 was given to a young woman who fell, through a defect in the sidewalk, and fractured her vertebra, so that paralysis ensued.

These citations are interesting, as showing what value courts and juries sometimes put upon bones and brains, and to urge upon us to discharge the duties of our profession with faithfulness. It has been decided by the courts that when a man uses dangerous means, and life is imperiled thereby, he cannot escape the consequences of his acts. Then the burden of proof must rest upon him to show that no other means could have been used, and if he showed a reasonable degree of care and skill in his treatment he cannot be held liable for the results; but to prove this he must have testimony from professional ranks. The plea that it was an accidental or innocent mistake will not avail him, and he will be liable, at the suit of the party injured, for damages at the discretion of the jury. Sound public policy in relation to the preservation of health and life would seem to require that this rule should have a rigid and inflexible application to cases when the laws of right treatment at the hands of any in our ranks should indicate the want of care and skill.

Instances may be quoted to show how important it is that we should have clear ideas upon this subject, where charges of improper liberties are made against operators, when anesthetics have been administered. You will know how often men have been unjustly accused of this, when the facts have proved beyond a question that there was not the slightest ground for them to rest upon. In these days of sudden deaths, when one can hardly read a daily paper without seeing one or more sudden deaths reported, has it not often occurred to you that such a case might occur in our operating chair, either with or without any anesthetic? I remember a case in my own practice, where a man of wonderful physical development came to the office to have a tooth extracted. He did not appear nervous at all. There was nothing in the operation to excite comment—a simple case of extraction—yet the moment that tooth left its socket that man became as rigid as marble—eyes set, stertorous breathing—it was some time before we got him around again. And so we will day by day have to take risks that we know not of, which may not manifest themselves, and yet in some unforeseen moment may overtake us. Dealing as we do with the nervous, the anti-nervous, the strong and the weak, it will readily occur to you why I decided in response to your invitation to read a paper to-night, to take the subject that I did.

I have tried to bring out some of the many points and reasons why we should have this matter written up and discussed. That the disease we are called upon to treat proceeds from pathological causes, and that lesions in the teeth and oral cavity are far-reaching, and go to sum up the happiness of the human family, and tend to their well being. We have, I think, sufficient ground for the necessity of a dental jurisprudence on which much remains to be written. The design of this paper will have accomplished its mission if what I have said will attract your attention to the importance of the subject, and lead to its investigation.

